# Detection of G1138A Mutation of the *FGFR3* Gene in Tooth Material from a 180-Year-Old Museological Achondroplastic Skeleton

**DOI:** 10.3390/genes8090214

**Published:** 2017-08-29

**Authors:** Lucas L. Boer, Jana Naue, Laurens de Rooy, Roelof-Jan Oostra

**Affiliations:** 1 Department of Anatomy and Museum for Anatomy and Pathology, Radboud University Medical Centre, Geert Grooteplein Noord 21, 6525 EZ Nijmegen, The Netherlands; lucas.boer@radboudumc.nl; 2 Swammerdam Institute for Life Sciences, University of Amsterdam, Science Park 904, 1098 XH Amsterdam, The Netherlands; j.naue@uva.nl; 3 Department of Medical Biology, Sections Clinical Anatomy & Embryology and Museum Vrolik, Academic Medical Center, University of Amsterdam, Meibergdreef 15, 1105 AZ Amsterdam, The Netherlands; l.derooy@amc.uva.nl

**Keywords:** achondroplasia, DNA analysis, *FGFR3*, G1138A, museum, skeletal dysplasias, teratology

## Abstract

Throughout the last four centuries, many anatomical museums across the world have collected teratological specimens that became precious objects. These can be regarded as spirits of the past which have captured the morphology of diseases through time. These valuable and irreplaceable specimens can be perfectly used in contemporary dysmorphological or genetic research. Unfortunately, due to the historical nature of these specimens and the regularly used aggressive preservation fluids, DNA degradation is often present. Furthermore, the use of material for DNA extraction is restricted to preserve the appearance of these valuable museological specimens. Thus, the most challenging part in this perspective is to harvest sufficient DNA of good quality for further testing without damaging the specimens. Besides fixated specimens, most teratological collections contain dried skeletal and teeth materials which are an excellent source to extract DNA. We here present a DNA-based method that enables genetic identification of the G1138A mutation of the *FGFR3* gene in a 180-year-old achondroplastic skeleton, confirming the previously morphologically determined disease. Nuclear DNA was extracted from a premolar tooth and the mutation was found using Sanger sequencing of a small region of the *FGFR3* gene.

## 1. Introduction

Many anatomical museums throughout the world contain, maintain, and exhibit teratological collections. These predominantly historical accumulations of nature’s unpredictability can be seen as irreplaceable treasure chests waiting to be explored with contemporary (dys)morphological knowledge and supplementary genetic research. Although some museums perform additional re-describing and re-diagnosing of their teratological specimens, this is still quite exceptional, taking into account the many existing teratological collections (e.g., [1,2,3,4]). Therefore, many exhibited specimens have never been viewed from a modern dysmorphological stance, let alone investigated with additional diagnostic techniques. Nevertheless, most of the historically made diagnoses, which were based on contemporary classifications of diseases and ideas of embryological development, do not fit modern medical and biological standards. 

Only a limited number of inherited disorders may be morphologically revealed in the skeleton, of which achondroplasia is an example. Achondroplasia (OMIM100800) is a fully penetrant autosomal dominant Mendelian disorder and is considered one of the most common forms of short-limb dwarfism in humans [5]. It occurs with a frequency of one in 10–30,000 births [6], and in 90% of the cases it concerns a de novo mutation which is strongly correlated with high paternal age [7]. Homozygous inherited forms of achondroplasia are lethal in early infancy due to small thoracic cage size and thereby respiratory distress [8]. 

Achondroplasia was initially described by Parrot in 1876 [9]. In addition to being a misnomer in itself (see further), it inappropriately became a receptacle for almost any short-limbed skeletal dysplasia, leading to many misdiagnoses. With increasing genetic diagnostic sophistication, many of these disorders were recognized as entities of their own. 

**Figure 1:**
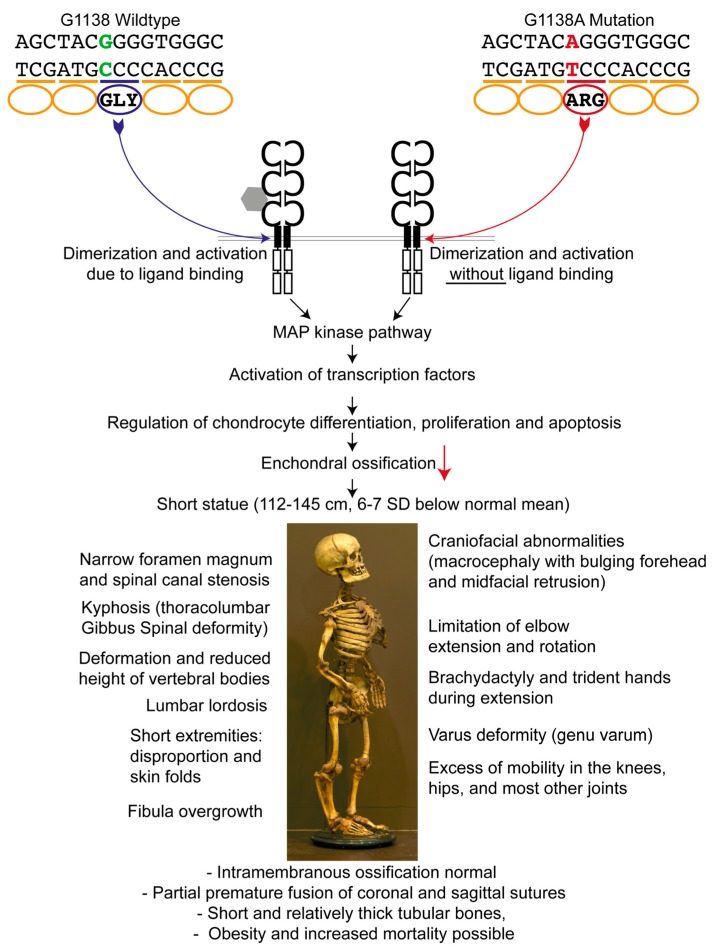
Genetic background and consequences of the fibroblast growth factor receptor-3 gene (FGFR3) mutation. The G1138A mutation leads to an amino acid exchange within the transmembrane region of FGFR3, resulting in a ligand-independent activation of the downstream pathways. FGFR3 is a negative regulator of enchondral ossification. In the case of a mutation, increased protein activity and thereby dramatically decreased enchondral ossification with normal intramembranous ossification leads to the observed anomalies in achondroplasia [9,16,17,18,19,20,21,22]. SD: standard deviation.

In 1994, the gene responsible for achondroplasia was obtained by linkage analysis and mapped to a 2.5 Mb of DNA located at the telomeric region of the short arm of human chromosome 4 (4p16.3) containing the fibroblast growth factor receptor-3 gene (*FGFR3*) [10,11]. A paper by Etlik et al. [12] reports that in 97% of the affected individuals a G-to-A transition, and in 1% a G-to-C transition at nucleotide 1138 (G1138A, G1138C) occurs in the *FGFR3* gene (dbSNP 150: rs28931614). This mutation results in the substitution of a glycine residue for an arginine residue (Gly380Arg) in the transmembrane domain of the FGFR3 protein [7] (Figure 1); in around 2% of cases, other positions within the gene are affected [13]. As a result of these mutations, FGFR3 can be activated without binding to fibroblast growth factors [14], and since *FGFR3* normally regulates chondrocyte differentiation, proliferation, and apoptosis, diminished diaphyseal growth ensues [15]. The growth plate cartilage is therefore dysplastic, although the name of the condition erroneously implies that the cartilage is absent. Achondroplasia is characterized by a markedly shortened stature. In males, it ranges between 118 cm and 145 cm, and in females between 112 cm and 136 cm, which is 6–7 standard deviations (SD) below the normal mean [16]. Other prominent features are rhizomelic shortened limbs, bulging forehead, and midfacial retrusion (Figure 1, bottom part). Like many other skeletal dysplasias, it mainly affects enchondrally rather than intramembranously ossifying skeletal elements.

Here, we report on the identification of the Gly380Arg mutation of the *FGFR3* gene from tooth material in a 180-year-old museological achondroplastic skeleton from Museum Vrolik, the anatomical museum of the the University of Amsterdam (The Netherlands). This skeleton was originally acquired before 1830 by Gerard Vrolik (1775–1859), founder of Museum Vrolikianum, the predecessor of the current Museum Vrolik.

Pusch et al. (2004) attempted a screening of ancient bone samples for achondroplasia mutations, but failed because of significant false positivity in their normal controls [23]. No other attempts have been reported that we know of. To the best of our knowledge, we are the first to unequivocally establish a genetic condition using DNA obtained from dried ancient tooth tissue.

## 2. Materials and Methods

### 2.1. Achondroplastic Skeleton

Specimen M717 (Figure 1) concerns a macerated and dried skeleton in which much of the cartilaginous parts and some ligaments have been preserved. According to the catalogue of the original Vrolik collection [24], the skeleton (at the time numbered D239) was that of a male “Prussian dwarf” with a postural length of 116 cm, a slight thoracic scoliosis, and short bowed limbs. This supposedly resulted from to rickets [24], a default diagnosis in the 19th century for virtually any condition that included bowing of tubular bones that did not obviously result from fractures. Upon external examination, a clear midfacial hypoplasia, a bell-shaped thorax, slight thoracic scoliosis, profound lumbosacral lordosis, cuboid shaped vertebrae, and a narrow pelvis with small iliac wings and flat acetabula can be seen. The tubular bones are small and robust with genu varum and relatively long fibulae. This particular specimen was, based on external dysmorphological characteristics, diagnosed with achondroplasia [3]. To verify this diagnosis, the left upper first premolar was extracted under the most careful conditions to prevent contamination with recent DNA. DNA from this tooth was obtained using the undermentioned protocol. For control purposes, a tooth (collected between 1930–1950) of a control individual was used. The Museum Vrolik collection comprises one other specimen of an adult achondroplastic skeleton (specimen M718) [3], which we also intended to sample. However, all teeth appeared to be glued in their sockets and we were unable to extract any of them without damaging the specimen.

### 2.2. Sample Preparation

**Figure 2:**
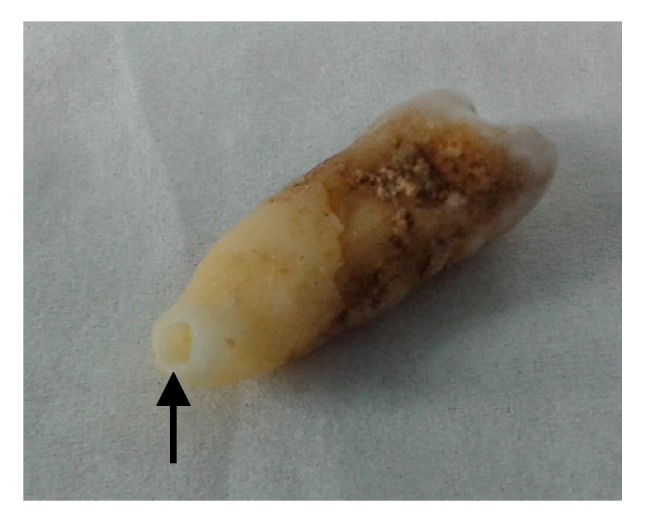
Photo of the tooth extracted from the achondroplastic skeleton. Powder from the premolar tooth was obtained by drilling into the tooth from the tip of the root (black arrow).

The whole teeth preparation and drilling was performed within a cleaned (bleach, UV-light) and closed environment under pre-polymerase chain reaction (PCR) conditions, using decontaminated equipment, human DNA-free material, and protective clothing (gloves, mouth, head, and arm protection), to allow human DNA-free working conditions. The following procedure was conducted in both the control tooth as well as in the tooth of the achondroplastic skeleton. To prevent loss of museological value of the specimens, the procedure aimed to obtain DNA-containing material in the least destructive way. Testing was performed independently to avoid cross-contamination between the two samples. The tooth was first decontaminated using a swab with bleach, followed by DNA-free water to mechanically clean it. Afterwards, the tooth was cleaned twice for 30 min in ethanol and once in DNA-free water for 30 min. The surface was further decontaminated by UV-light for 30 min. A hole was drilled from the tip in the root of the tooth (Figure 2) using a drilling machine (Sybron Endo), and a carbon drill (diameter: 1.6 mm, E0205, Dentsply LN Bur) (both from Henry Schein Dental, New York, NY, USA) with an operating speed of 300 rpm. Due to the low speed, fixation was done by hand, allowing precise handling. Drilling was performed until the pulp chamber was reached. All the obtained tooth powder was collected. In the case of the tooth acquired from the achondroplastic skeleton, drilling within the same hole was performed twice to obtain additional material for a second extraction. The powder amount was weighted with a micro scale. The powder originated mostly from dentine material, but also cementum (from the beginning of the drilling) and material from the pulp chamber surface was retained (from drilling within the chamber). The small hole in the root of the tooth was invisible after placing the element back into the skeleton. 

### 2.3. DNA Extraction

DNA extraction was performed in another pre-PCR room. For initial decalcification, 1 mL of 0.5 M EDTA (pH 8.0) was added to the tooth powder and incubated at 30 °C under rotation for 1 h. The decalcified material was incubated overnight at 56 °C using the lysis buffer of the Gene Matrix Bone DNA Purification Kit (EURx, Gdansk, Poland). DNA extraction was performed following the manufacturer’s recommendations. Final DNA elution was executed in 50 µL and 30 µL elution buffer, for the first and second DNA extraction, respectively. DNA concentration was measured using the Qubit dsDNA HS Assay Kit with the Qubit 3.0 fluoremeter (Thermo Fisher Scientific, Waltham, MA, USA). 

### 2.4. Initial PCR Testing for the Presence of DNA

PCR setups were performed within a specific pre-PCR hood, and PCR products handled in a post-PCR area. As degradation was expected, a sensitive Alu-PCR (71 bp fragment) was used to test whether an amplifiable amount of DNA was obtained. PCR was performed using the Yb8-F and Yb8-R primer according to Walker et al. [25]. The 10-µL reaction mix consisted of 5 µL of HotStarTaq Master Mix Kit (Qiagen, Hilden, Germany), 5 pmol of each primer, and 750 pg of DNA. PCR conditions were: 95 °C for 10 min, followed by 36 cycles of 30 s at 94 °C, 30 s at 60 °C, 30 s at 72 °C, and a final elongation for 10 min at 72 °C. The PCR product was verified on a gel.

### 2.5. Mutation Detection Using PCR and Sanger Sequencing

PCR of the area of interest around the positions with the expected mutation G1138A/C (rs28931614, chr4:1804392) and the less common mutation G1123T (rs75790268, chr4:1804377) was performed using a primer pair leading to a fragment of 164 bp according to Shiang et al. [26] (Forward (F): AGGAGCTGGTGGAGGCTGA, Reverse (R): GGAGATCTTGTGCACGGTGG). Another PCR using a different reverse primer (R1: CCAGGCCTTTCTTGGGGG) was performed to obtain a shorter fragment of 138 bp. The 10-µL reaction mix consisted of 5 µL of HotStarTaq Master Mix Kit (Qiagen), 5 pmol of each primer, 1 µL BSA (1 mg/mL) (Thermo Fisher Scientific), and between 1 ng and 15 ng of DNA. PCR was run under the following conditions: 95 °C for 10 min, followed by 36 cycles of 30 s at 94 °C, 30 s at 65 °C, 30 s at 72 °C, and a final elongation for 10 min at 72 °C. In addition, two more cycles were executed for the analysis of the second DNA extract to obtain a higher amount of PCR product for sequencing. Successful amplification was verified on gel, and the PCR product was cleaned with 10 U Exonuclease I and 1 U thermo-sensitive Alkaline Phosphatase (FastAP) (both from Thermo Fisher Scientific) for 30 min at 37 °C, which was followed by enzyme inactivation for 15 min at 85 °C. Cleaned PCR products were send to sequencing (Eurofins Genomics, Luxembourg, Luxembourg) using the reverse primers. A negative DNA extraction/PCR control and a positive PCR control (1 ng control DNA 9947A, Promega, Madison, WI, USA) were taken along, showing the expected results.

## 3. Results

### 3.1. Obtained Material for Analysis and Pre-Testing

Forty milligrams of dental powder were used for DNA extraction in the case of the control tooth, resulting in 270 ng DNA in total. In the case of the achondroplastic skeleton, 45 mg and 53 mg of dental powder were retrieved from the first and second drilling of the premolar, resulting in a total of 7.4 ng and 10.26 ng of DNA, respectively. Analysis of the first DNA extract using the Alu-PCR was successful, showing that amplifiable DNA was available from the tooth and that the DNA extraction was able to remove possible PCR inhibitors successfully.

### 3.2. FGFR3 Analysis for Mutation Detection

**Figure 3:**
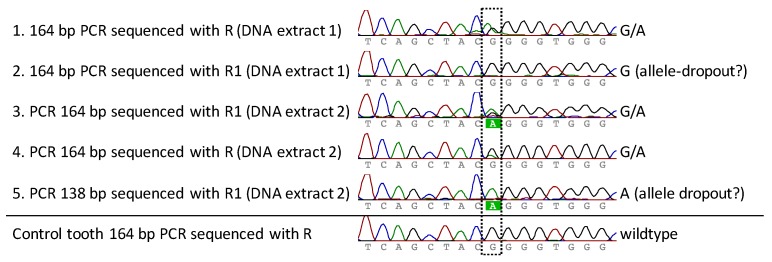
Sequencing results of multiple PCRs from the premolar of the achondroplastic skeleton, as well as the analysis of a control tooth. Rows 1–5: Two independent DNA extracts of the premolar were analyzed for the presence of a mutation. Two different long PCR-systems as well as two sequencing primers were used for analysis. In two PCRs, only the wildtype G or the mutant allele A was observed, indicating allelic-dropout as the G/A was detected in the other three PCRs. The analyzed control tooth resulted in the expected wildtype.

*FGFR3* PCR and sequencing analysis of both DNA extracts of the premolar was performed multiple times for both PCR systems (164 bp and 138 bp) in order to obtain reproducible and reliable results (success rate receiving good sequences: first DNA extraction: 50% (3/6 analysis), second DNA extraction: 75% (3/4 analysis)). The 138 bp system was only tested in total three times, as a lower amplification efficiency was known from pre-experiments. Additionally, an analysis was performed for the control tooth to exclude PCR artifacts. All successful sequence results covering the position of interest are presented in Figure 3 (excluding the PCR and F1 sequence reaction not covering the position in good quality). A heterozygote G-to-A mutation at position chr4:1804392 was observed, which corresponds to the expected G1138A (rs28931614) exchange within the *FGFR3* gene. Two sequencing reactions resulted in only the wild-type (G) or the mutant (A) nucleotide (Figure 3, rows 2 and 5), which we assume was caused by allelic-dropout during the the PCR reaction. Multiple analysis using two different PCR systems, two different sequencing primers, as well as independent analysis with the second DNA extract confirmed the presence of the heterozygous G1138A mutation. The analysis of the control tooth resulted in the detection of the expected wildtype sequence and gave no indication for PCR artifacts.

## 4. Discussion

Old museological collections can be used eminently for further explorations regarding their excellent scientific potential. Research of old teratological specimens can yield valuable contributions in e.g., etiopathogenetic issues and potentially expand the literature beyond the restrictions of single case studies due to the plurality of specimens with similar anomalies in these collections [27]. One should, however, avoid focusing the policies regarding the management and study of these collections entirely on medical and biological aspects, thereby neglecting the irreplaceable value that these collections have as cultural-historical objects. Without being aware of the latter, researching and handling these collections may cause irreversible damage to the historical integrity of these specimens and preparations.

Many historical anatomical and pathological collections in the world have already lost much information due to the unawareness of this historical and cultural aspect. In this study, the actual extraction of the tooth from the historical skeleton was therefore performed and supervised by museum specialists, making sure that the damage done to the specimen was limited to the minimum.

In spite of the often beautiful and elegant appearance of these old teratological specimens, their age and frequently used formaldehyde-based preservation fluids dramatically affect the integrity of the DNA quality to the point that it is unsuitable for additional genetic testing. Nevertheless, although DNA extraction from formalin-fixated and paraffin-embedded specimens is often accompanied with poor results, analysis of gene products and/or their metabolic effects can yield valuable contributions in the verification of assumed genetic diagnoses [28]. 

Another possible source where DNA can be retrieved from is the many macerated skeletons which are present in most historical collections. These skeletons were presumably only mildly macerated until all fleshy parts could be scraped off; hence the ligaments and cartilage would be retained by this delicate procedure. After the skeletons were dried, they became solid, natural positioned, infrangible time witnesses of nature’s capriciousness. Due to the nature of this relatively mild cleaning process and the solidness of these skeletons through time, DNA can still reside in both bone and teeth [29]. 

We managed to successfully extract nuclear DNA from dental material originating from a 180-year-old achondroplastic skeleton from Museum Vrolik in Amsterdam, The Netherlands. The original museum collection was privately owned and was further expanded by Gerard Vrolik’s eldest son Willem Vrolik (1801–1863). When Willem Vrolik died, the collection comprised of 5123 anatomical preparations [30]. The analysis of ancient human DNA material is challenging due to multiple factors. Firstly, exogenous DNA contamination introduced during the process needs to be avoided. Therefore, surface cleaning as well as handling under DNA-free conditions are essential. Secondly, the museological value of old specimens—which should stay intact—restricts possible sites of material extraction. Therefore, drilling through the tip of a tooth root is a good compromise. Using a molar instead of a premolar would have been favorable to obtain more DNA material, but was not possible due to the fixation of these teeth to the jaws. Non-destructive methods have been described previously, but they may change the tooth surface and cause increased porosity or lighter appearance [31,32,33]. Hence, we did not use this way of DNA extraction, as these consequences were not foreseeable. The third challenge is the analyzed material itself. Preservation conditions as well as the age lead to DNA degradation and cross-linking, in the case of formalin-fixated material. The use of bone material, and especially teeth, are known to be a good (and often the only available) source for DNA extraction [34]. However, the DNA quantity we obtained was small, which was also caused by the fact that we reduced the destruction of the tooth to a minimum, using only a small amount of dental material. Nevertheless, around 40 to 50 mg was enough for a successful analysis. To overcome the DNA degradation problem, only a small 138 bp and a 164 bp DNA region were amplified. Furthermore, the drilling speed was reduced to 300 rpm in order to avoid additional degradation of the DNA due to heat production. That effect was also seen by Adler et al. (2011), comparing mtDNA analysis after 1000 rpm and 100 rpm drilling [35]. 

PCR analysis confirmed the presence of the heterozygote G1138A mutation, which is pathognomonic for achondroplasia. The detection of the heterozygote version stands in accordance with the fact that the homozygous version would be lethal within early infancy. Furthermore, precautions to avoid any contamination were taken; therefore, we have no indication that the wild-type G was caused by any external DNA. We also do not see any indication that our results are caused by PCR artifacts, as analysis of the control tooth resulted in the expected wild-type. As mentioned before, we know of only one study by Pusch et al. (2004) in which ancient DNA material obtained from a 7000-year-old Egyptian mummy was investigated for the presence of a mutation causing achondroplasia, but they experienced false-positive PCR results in their control material [23]. Extract-induced PCR sequence changes were proposed as the most reasonable explanation, and other reasons, such as deamination processes over time, were excluded [23]. We did not see such a result in our control material. Also, no other sequence alterations in the achondroplastic skeleton were detected. However, the storage time of our control material (67–87 years) and that of the achondroplastic skeleton (180 years) is far shorter compared to the examined mummies in Pusch’s report, and the materials we used were preserved under good conditions. Furthermore, a column-based extraction method was used, which removes additional compounds very well, decreasing the risk of extract-induced sequence changes. The use of two DNA extracts, two PCR products, and two different sequencing primers demonstrated the reliability of the analysis. Unfortunately, the use of the forward primer was disadvantageous due to its close location to the position of the expected mutation. The sequencing result indicated the presence of the mutation; however, the quality of the sequence was not sufficient for a reliable base call. 

DNA analysis from ancient material is often performed in forensic cases for identification purposes or in archeological investigations. The analysis of diseases is a rarer event; however, the molecular method presented here opens new doors to verify diagnoses of museum specimens or other skeletal materials which are often based on external dysmorphological descriptions only.
